# Meta-analysis: loop ileostomy versus colostomy to prevent complications of anterior resection for rectal cancer

**DOI:** 10.1007/s00384-024-04639-2

**Published:** 2024-05-08

**Authors:** Shilai Yang, Gang Tang, Yudi Zhang, Zhengqiang Wei, Donglin Du

**Affiliations:** 1 Department of Gastrointestinal Surgery, The First Affiliated Hospital of Chongqing Medical University, Chongqing, China; 2 Division of Biliary Tract Surgery, Department of General Surgery, West China Hospital, Sichuan University, Chengdu, Sichuan China; 3 College of Combination of Chinese and Western Medicine, Chongqing College of Traditional Chinese Medicine, No. 61, Puguobao Road, Bicheng Street, Bishan District, Chongqing, 402760 P.R. China

**Keywords:** Loop colostomy, Loop ileostomy, Anastomotic leakage, Colorectal cancer

## Abstract

**Purpose:**

Anastomotic leakage is a serious complication of colorectal cancer surgery, prolonging hospital stays and impacting patient prognosis. Preventive colostomy is required in patients at risk of anastomotic fistulas. However, it remains unclear whether the commonly used loop colostomy(LC) or loop ileostomy(LI) can reduce the complications of colorectal surgery. This study aims to compare perioperative morbidities associated with LC and LI following anterior rectal cancer resection, including LC and LI reversal.

**Methods:**

In this meta-analysis, the Embase, Web of Science, Scopus, PubMed, and Cochrane Library databases were searched for prospective cohort studies, retrospective cohort studies, and randomized controlled trials (RCTs) on perioperative morbidity during stoma development and reversal up to July 2023, The meta-analysis included 10 trials with 2036 individuals (2 RCTs and 8 cohorts).

**Results:**

No significant differences in morbidity, mortality, or stoma-related issues were found between the LI and LC groups after anterior resection surgery. However, patients in the LC group exhibited higher rates of stoma prolapse (RR: 0.39; 95%CI: 0.19–0.82; P = 0.01), retraction (RR: 0.45; 95%CI: 0.29–0.71; P < 0.01), surgical site infection (RR: 0.52; 95%CI: 0.27–1.00; P = 0.05) and incisional hernias (RR: 0.53; 95%CI: 0.32–0.89; P = 0.02) after stoma closure compared to those in the LI group. Conversely, the LI group showed higher rates of dehydration or electrolyte imbalances(RR: 2.98; 95%CI: 1.51–5.89; P < 0.01), high-output(RR: 6.17; 95%CI: 1.24–30.64; P = 0.03), and renal insufficiency post-surgery(RR: 2.51; 95%CI: 1.01–6.27; P = 0.05).

**Conclusion:**

Our study strongly recommends a preventive LI for anterior resection due to rectal cancer. However, ileostomy is more likely to result in dehydration, renal insufficiency, and intestinal obstruction. More multicenter RCTs are needed to corroborate this.

## Introduction

Colorectal cancer accounts for approximately 10% of newly diagnosed cancers and cancer-related mortalities annually worldwide. Additionally, it is the third most common cancer in men; and the second most common cancer in women worldwide [1, 2]. Three-quarters of all colorectal cancer cases reported are located in the rectal region [3]. Heald’s surgical concept of total mesorectal excision (TME) is the mainly adopted routine surgical dissection technique that reduces the local recurrence rate and improves the surgical rates of sphincter-preserving function in rectal cancer. However, anastomotic leakage (AL) remains one of the most frequent complications encountered in anterior resection procedures for all rectal cancer cases [4–6].

Previous reports have confirmed that up to 20% of patients experience AL after undergoing a low or ultralow anterior resection (LAR or uLAR) for rectal cancer [7, 8]. AL is directly linked to local recurrence and a reduction in the overall survival (OS) rate of patients with rectal cancer [9–11]. Therefore, AL is a major concern for surgeons who are now prioritizing finding the best solution for reducing this complication in rectal cancer management. A diverting stoma has been shown to significantly lower the risk of anastomotic leakage, thereby reducing the outcomes of AL [12]. Patients with rectal cancer who are at a higher risk of AL are those with advanced age, obesity, cardiovascular comorbidities, concurrent corticosteroid use, bowel obstruction, neoadjuvant chemoradiotherapy, and a shorter tumor distance from the anal verge. These high risk patients who undergo an anterior resection must have a temporary diverting stoma to prevent AL [13, 14]. However, the choice of stoma technique remains debatable.

The most popular stoma options are the transverse LC or LI. Currently, most surgeons perform a temporary ileostomy. The overall complications of ileostomy are fewer than those of colostomy, and the procedure is simple. With the increasing awareness of the complications of ostomy and the improvement of surgical techniques, some articles tend to favor colostomy. A recent study showed that the overall complication rate was significantly higher in the ileostomy group than in the colostomy group. This is particularly the case with regard to ostomy prolapse, contrary to many previous studies [15]. There is also literature that shows no difference in overall complications between the two [16]. LC and LI, for different populations, the selection of a suitable stoma is key, and the unique complications of LC and LI require further clarification. This study will compile and evaluate the completed trials of LI and LC. Subsequently, updated criteria and continuous meta-analysis will be used to determine the optimal approach.

## Methods

This meta-analysis was conducted following the Preferred Reporting Items for Systematic Reviews and Meta-Analyses (PRISMA) guidelines [17]. The study protocol was registered in PROSPERO (CRD42024522102).

## Data collection

**Fig. 1:**
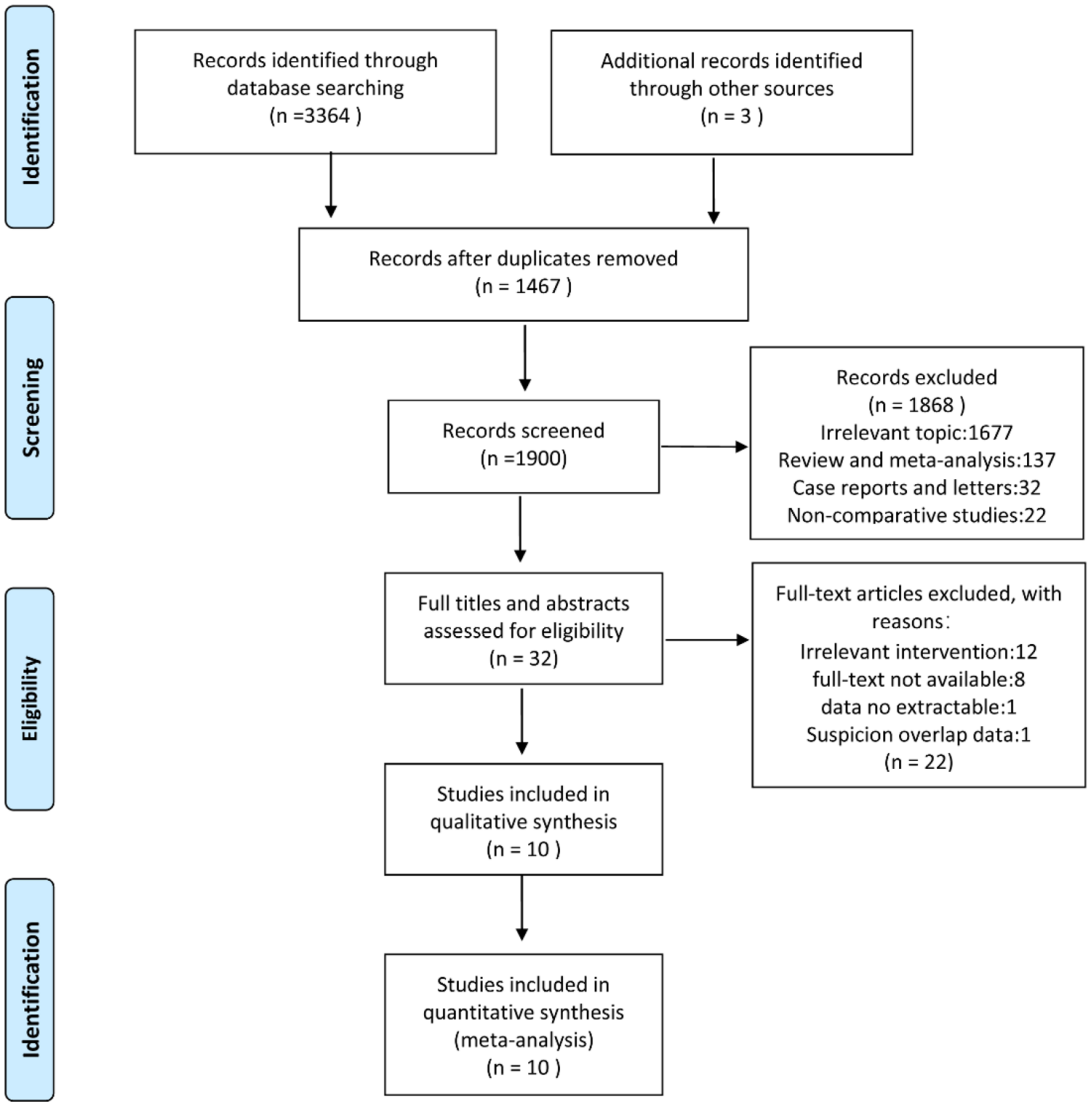
Flow chart of literature search and screening

From inception to July 2023, searches were performed in the databases of Embase, Web of Science, Scopus, PubMed, and the Cochrane Library using the keywords: "ileostomy," "colostomy," "rectal cancer," "rectal carcinoma," and "rectal cancer". Figure 1 presents a summary of the search approach.

## Criteria for inclusion and exclusion

Inclusion criteria:

- Loop ileostomy or loop colostomy performed during anterior resection in studies involving individuals with colorectal cancer
- Studies designed as cohort studies or randomized controlled trials.

Exclusion criteria:

- Studies involving diverticulitis and other diseases;
- Loop ileostomy or loop colostomy not performed simultaneously during anterior resection;
- Abstracts from meetings, correspondence, reviews, investigations with non-human participants, and case studies
- Trails with duplicate data, such as the same institutional data or overlapping data were excluded.

## Study selection

Two review writers independently assessed the abstracts and titles of potential studies to select studies that met our inclusion criteria. The full texts of the papers, which may be of interest, were obtained. The authors identified studies that met the inclusion criteria through independent evaluation of full-text records. Any disagreements regarding study selection were resolved by consensus and discussion among our author group.

## Data extraction

Three assessors independently extracted information from the eligible studies, including the author names, publication year, country, sample size, and study duration. Outcome measures included morbidity, mortality, AL and complications associated with stoma creation, such as stoma-prolapse or retraction, stoma-stricture, stoma-bleeding, stoma edema, parastomal dermatitis, parastomal hernia, parastomal infection or sepsis, high output, and renal insufficiency during stoma creation. During stoma closure, the following complications may occur: morbidity, mortality, anastomotic fistula, surgical site infection, incisional hernia, ileus, and the time from operation to first defection and discharge.

## Quality assessment

The risk of bias in the RCTs was evaluated using the Cochrane Collaboration tool for risk of bias, which covers the following domains: (a) sequence generation; (b) allocation concealment; (c) participant and staff blinding; (d) blinding of outcome assessment; (e) incomplete outcome data; (f) selective outcome reporting; and (g) additional possible sources of bias. The Newcastle-Ottawa scale (NOS) was used to evaluate the potential for bias in the cohort research. Nine points were allocated for each of the three methodological components evaluated: result, group comparability, and participant selection. Any discrepancies between the three writers (Tang, Du, and Yang) were discussed and solved with the fourth author (Wei) throughout the literature retrieval, screening, information extraction, and quality evaluation processes.

## Statistical analysis

The risk ratio (RR) and 95% confidence intervals (CIs) were calculated. For continuous outcome data, the mean difference (MD) and associated 95% CI were computed. Cochran's Q test statistic was used to assess the heterogeneity of the studies. Given the possibility of methodological and clinical heterogeneity, the random-effects model was used in all quantitative analyses. Review Manager (RevMan) Version 5.3 (The Nordic Cochrane Centre, The Cochrane Collaboration 2014; Copenhagen, Denmark) was used for the analyses. Funnel plot analysis was conducted to assess the publication bias. Statistical significance was set as P < 0.05.

## Results

In this study, 3367 articles were initially identified. After removing 1467 duplicate entries, 1900 articles were eliminated based on evaluation of the title and abstract of each article. Subsequently, the remaining 32 papers underwent full-text examination, resulting in the identification of 10 articles that the inclusion criteria for analysis [15, 16, 18–25] (Fig. 1). This meta-analysis comprised 10 trials involving 2036 participants from six countries (China, France, Norway, Turkey, England, and Germany). Table 1 provides an overview of the features of the included studies. Among the ten qualifying studies, there were two RCTs, one prospective cohort study, and seven retrospective cohort studies.

**Table 1:**
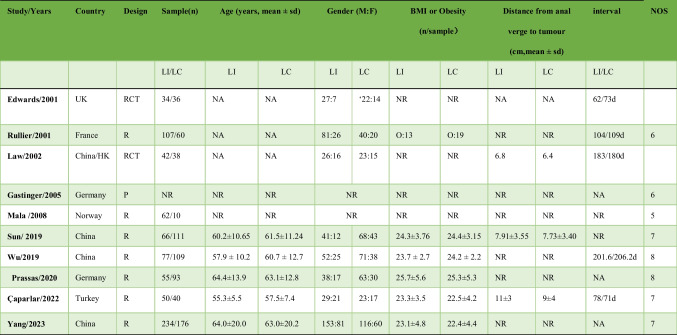
The basic characteristics of included studies

*O* Obesity, *Rct* Randomized controlled trial, *P* Prospective cohort study, *R* Retrospective cohort study, *NR* Not reported, *NA* No significance

## Quality assessment

**Fig. 2:**
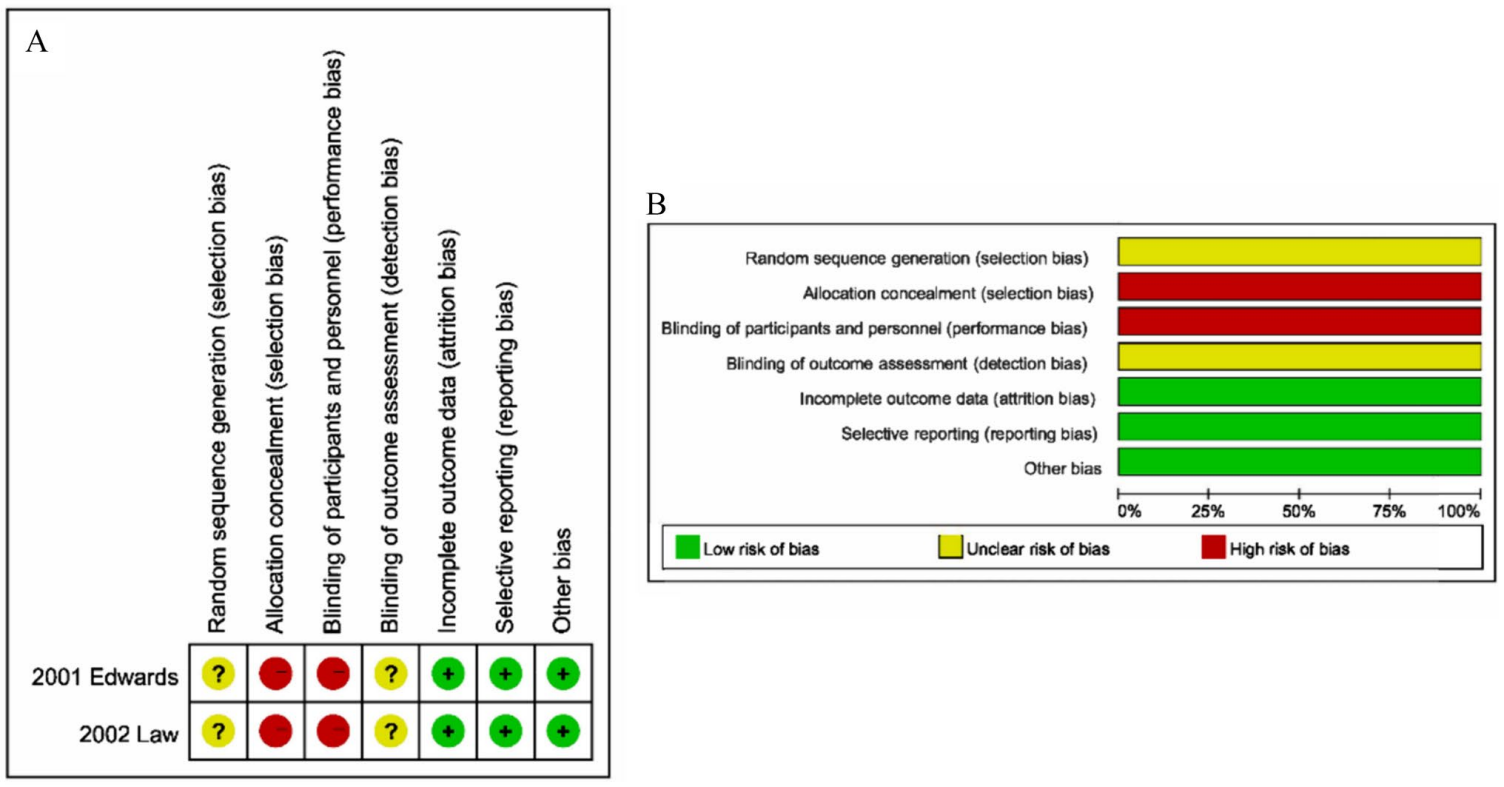
Risk of bias for each included RCT study. **A** Risk of bias summary. **B** Risk of bias graph

Eight trials scored five or higher on the NOS, indicating good quality (Table 1). Figure 2 illustrates the low risk of bias observed in the RCTs.

## Outcomes

### Complications of stoma

#### Morbidity and mortality following stoma development

**Fig. 3:**
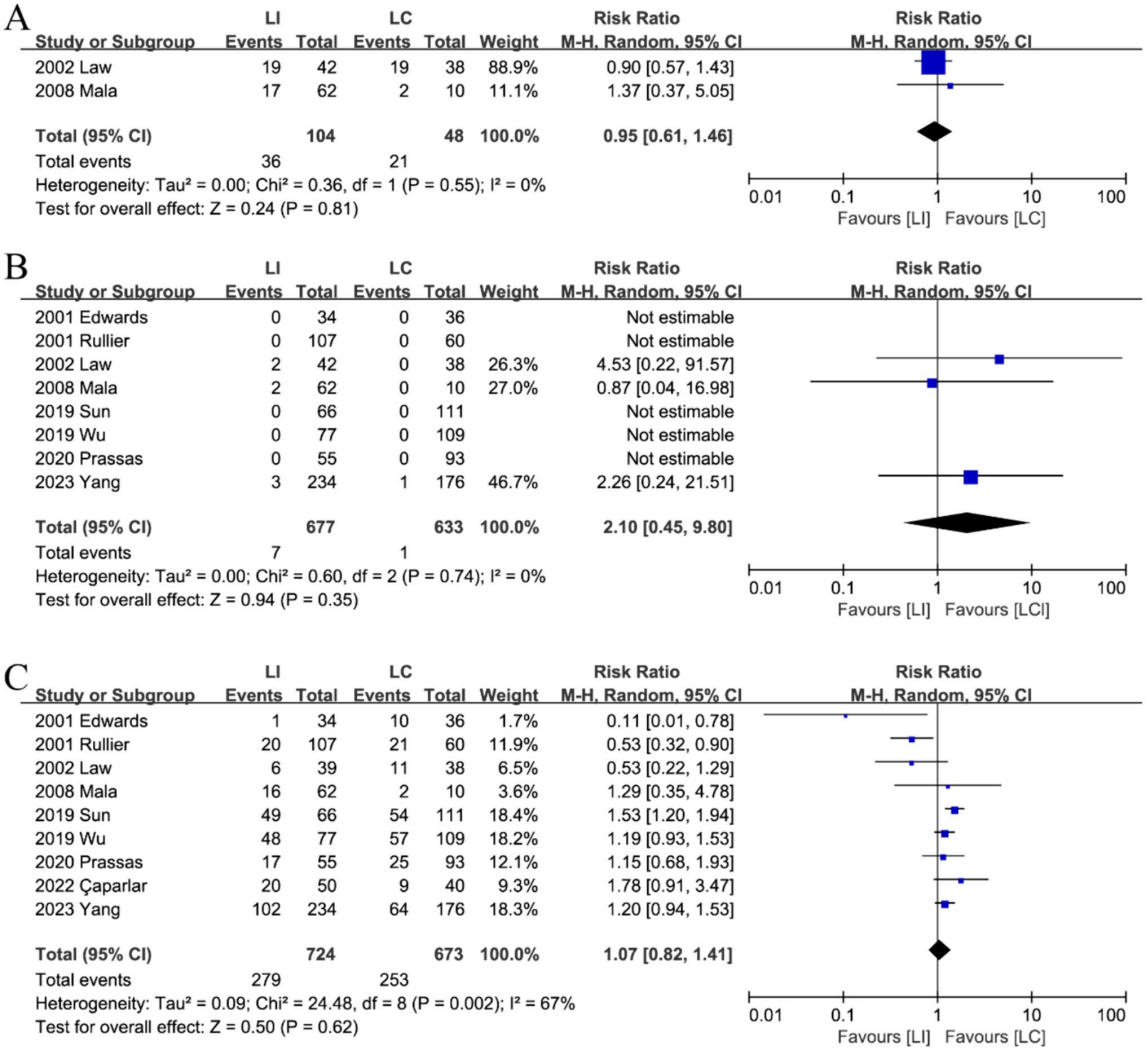
The morbidity (**A**), mortality (**B**) and stoma-related complication (**C**) in the stoma formation between LI and LC groups

Eight studies [15, 16, 18–21, 23, 24] evaluated mortality between the preventive LI and LC groups, while two researchers assessed morbidity [20, 24]. There was no significant difference observed in morbidity (RR: 0.95; 95%CI: 0.61–1.46; P = 0.81, Fig. 3A) or mortality (RR: 2.10; 95%CI: 0.45–9.80; P = 0.35, Fig. 3B). Furthermore, no heterogeneity was detected among the studies (Tables 2 and 3).

**Table 2:** Outcomes related to stoma formation

	LI(events/total, n)	LC(events/total, n)	Statistical method, RR	Estimated effect, 95%CI	I^2^
Morbidity following stoma development	36/104	21/48	0.95	0.61–1.46	0%
Mortality following stoma development	7/677	1/633	2.10	0.45–9.80	0%
Stoma-related complication	279/724	253/673	1.07	0.82–1.41	67%
Stoma prolapse	9/612	25/623	0.39	0.19–0.82	0%
Stoma retraction	25/628	46/627	0.45	0.29–0.71	0%
Stoma stricture	2/173	3/171	0.79	0.03–19.53	64%
Stome bleeding and edema	10/512	10/480	1.20	0.50–2.88	0%
Parastomal dermatitis	120/628	73/627	1.63	0.95–2.82	65%
Parastomal hernia	39/724	64/673	0.77	0.47–1.26	17%
Necrosis	1/184	2/169	0.52	0.06–4.18	0%
Parastomal infection & sepsis	7/407	11/347	0.60	0.17–2.16	41%
Dehydration or Electrolyte disturbance	27/362	9/330	2.98	1.51–5.89	0%
High-output	29/357	0/290	6.17	1.24–30.64	9%
Renal insufficiency	15/366	6/378	2.51	1.01–6.27	0%

*LI* Loop ileostomy, *LC* Loop colostomy, *CI* Confidence interval, *RR* Risk ratio

**Table 3:** Outcomes related to stoma closure

	LI(events/total, n)	LC(events/total, n)	Statistical method, RR	Estimated effect, 95%CI	I2
Morbidity after stoma reversal	151/691	76/421	1.15	0.77–1.72	39%
Mortality after stoma reversal	5/691	1/421	1.23	0.23–6.64	0%
Stoma closure-related complication	173/964	138/785	1.03	0.68–1.55	65%
Anastomotic fistula	15/803	6/642	1.35	0.46–4.01	11%
Surgical site infections (SSI)	32/642	60/597	0.52	0.27–1.00	41%
Incisional hernia	22/554	44/549	0.53	0.32–0.89	0%
Ileus	48/794	20/596	1.59	0.94–2.69	1%

*LI* Loop ileostomy, *LC* Loop colostomy, *CI* Confidence interval, *RR* Risk ratio

#### Stoma-related complications

Nine studies [15, 16, 18–21, 23–25] identified stoma-related problems and demonstrated no statistically significant difference between the two groups (RR: 1.07; 95%CI: 0.82–1.41; P = 0.62). Significant heterogeneity was observed (P < 0.01; I^2^ = 67%) (Fig. 3C).

#### Stoma prolapse

**Fig. 4:**
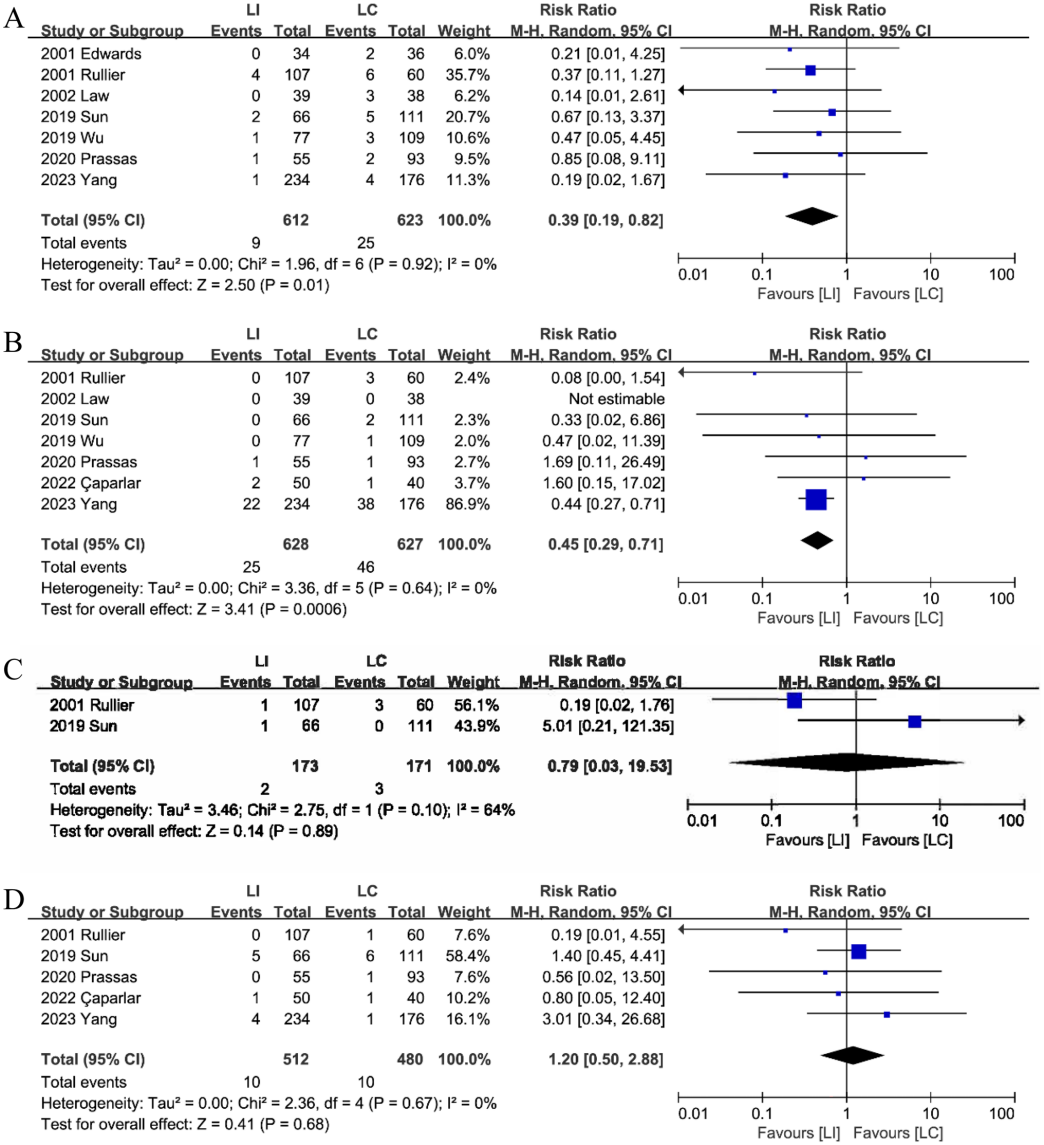
The stoma prolapse (**A**), stoma retraction (**B**), stoma stricture (**C**), and stoma bleeding and edema (**D**) between LI and LC groups

Seven studies [15, 16, 18–21, 23], concluded that LI reduces the incidence of stoma prolapse (RR: 0.39; 95%CI: 0.19–0.82; P = 0.01). The incidence of stoma prolapse was significantly lower in the LI group (1.5%, 9/612) than in the LC group (4.0%, 25/623). There was no discernible heterogeneity between the two groups. (P = 0.92, I^2^ = 0%) (Fig. 4A).

#### Stoma retraction

Seven studies [15, 16, 18–21, 25] reported data of stoma retraction and a comprehensive analysis revealed that stoma stricture was significantly lower in the LI group (4.0%,25/628)compared to the LC group (7.3%,46/627), with no observed heterogeneity (RR: 0.45; 95%CI: 0.29–0.71; P < 0.01; heterogeneity: P = 0.64, I^2^ = 0%)(Fig. 4B).

#### Stoma stricture

A comprehensive analysis of two studies [15, 19] reporting on stoma stricture showed no significant difference in stoma stricture between the LI and LC groups (RR: 0.79; 95%CI: 0.03–19.53; P = 0.89; heterogeneity: P = 0.1, I^2^ = 64%) (Fig. 4C).

#### Stoma bleeding and edema

No discernible difference in stoma hemorrhage and edema between the LI and LC groups was observed in five investigations [15, 16, 18, 19, 25]. (RR: 1.20; 95%CI: 0.50–2.88; P = 0.68; heterogeneity: P = 0.67, I^2^ = 0%) (Fig. 4D).

#### Parastomal dermatitis

**Fig. 5:**
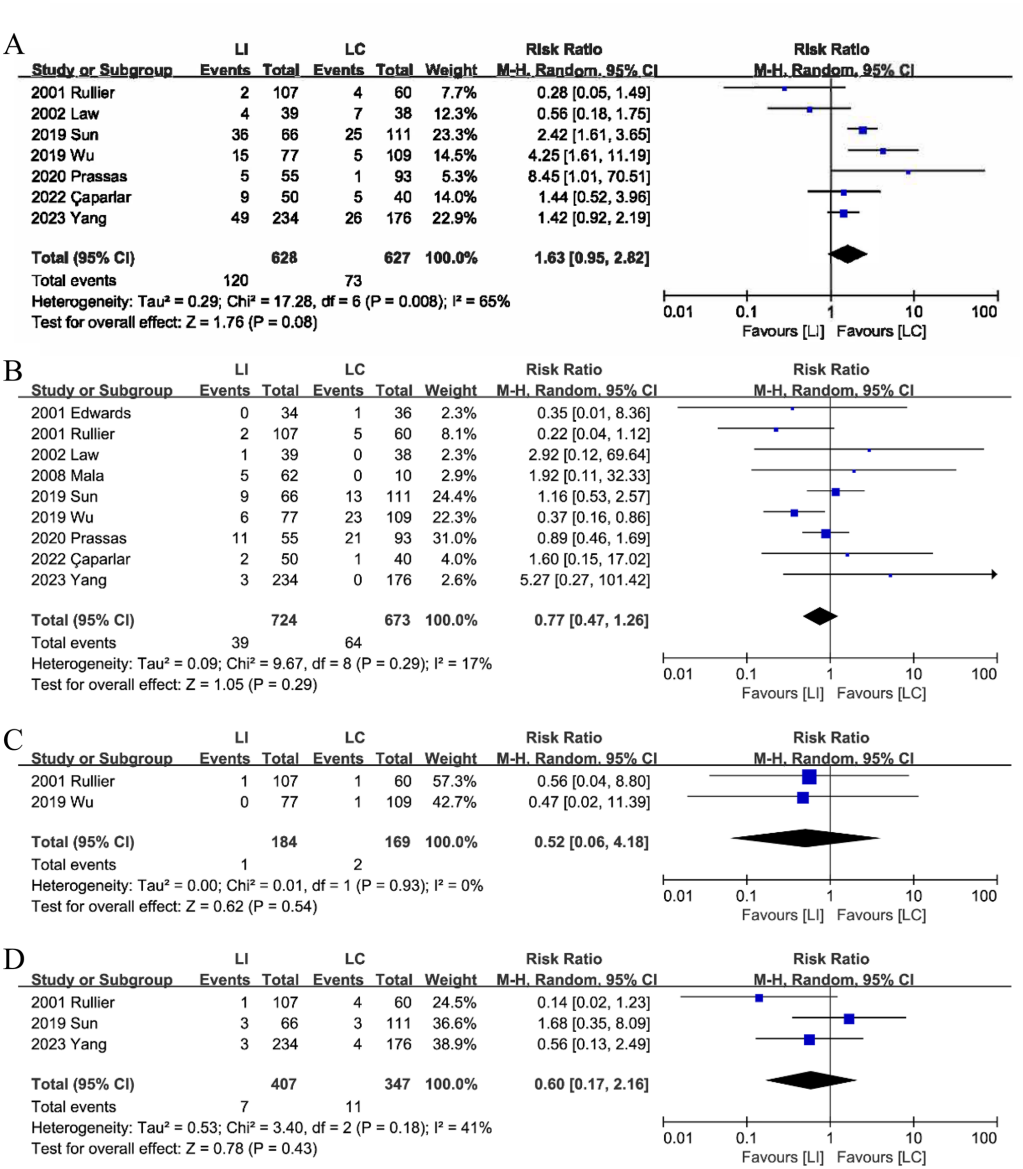
The parastomal dermatitis (**A**), parastomal hernia (**B**), necrosis (**C**), parastomal infection & sepsis (**D**) between LI and LC groups

Combined analysis from seven studies [15, 16, 18–21, 25] indicated that parastomal dermatitis did not significantly differ between the LI and LC groups, although the LI group showed a trend toward have more parastomal dermatitis than the LC group (RR: 1.63; 95%CI: 0.95–2.82; P = 0.08; heterogeneity: P = 0.008, I^2^ = 65%) (Fig. 5A).

#### Parastomal hernia

In the nine studies [15, 16, 18–21, 23–25] regarding parastomal hernia, no discernible difference was observed between the LI and LC groups (RR: 0.77; 95%CI: 0.47–1.26; P = 0.29; heterogeneity: P = 0.29, I^2^ = 17%) (Fig. 5B).

#### Necrosis

Comprehensive analysis revealed no significant difference in stoma necrosis between the LI and LC groups (RR: 0.52; 95%CI: 0.06–4.18; P = 0.54; heterogeneity: P = 0.93, I^2^ = 0%) (Fig. 5C). Two studies reported stoma necrosis [19, 21].

#### Parastomal infection and sepsis

Analysis from three studies [15, 18, 19] indicated that parastomal infection & sepsis did not significantly differ between LI and LC groups (RR: 0.60; 95%CI: 0.17–2.16; P = 0.43; heterogeneity: P = 0.18, I^2^ = 41%) (Fig. 5D).

#### Dehydration or electrolyte disturbance

**Fig. 6:**
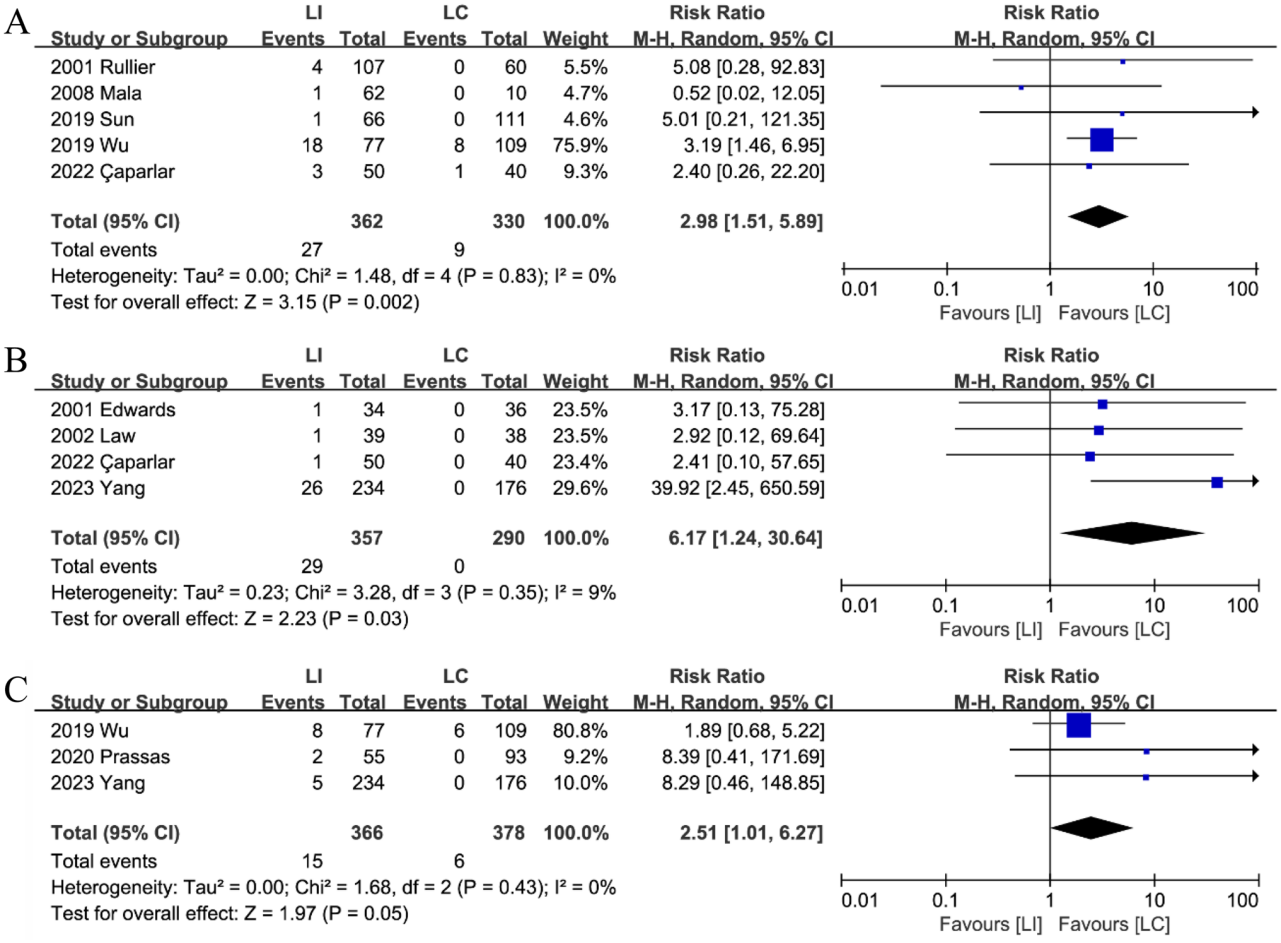
The Dehydration or Electrolyte disturbance (**A**), High-output (**B**) and renal insufficiency (**C**) between LI and LC groups

In each of the five trials [15, 19, 21, 24, 25], the LI group had a higher incidence of dehydration or electrolyte disruption than the LC group, with no heterogeneity observed. (Fig. 6A): (RR: 2.98; 95%CI: 1.51–5.89; P < 0.01; heterogeneity: P = 0.83, I^2^ = 0%).

#### High-output

A combination of four studies [18, 20, 23, 25] suggests that the LI group (8.1%,29/357) had a higher incidence of high-out stomas compared to the LC group (0%, 0/290). (RR: 6.17; 95%CI: 1.24–30.64; P = 0.03; heterogeneity: P = 0.35, I^2^ = 9%) (Fig. 6B).

#### Renal insufficiency

A combination of three studies [16, 18, 21] indicated that patients from the LI group (4.1%, 15/366) were more likely to experience renal insufficiency compared to patients from the LC group (1.6%,6/378) (RR: 2.51; 95%CI: 1.01–6.27; P = 0.05; heterogeneity: P = 0.43, I^2^ = 0%) (Fig. 6C).

### Complications following stoma closure

#### Morbidity after stoma reversal

**Fig. 7:**
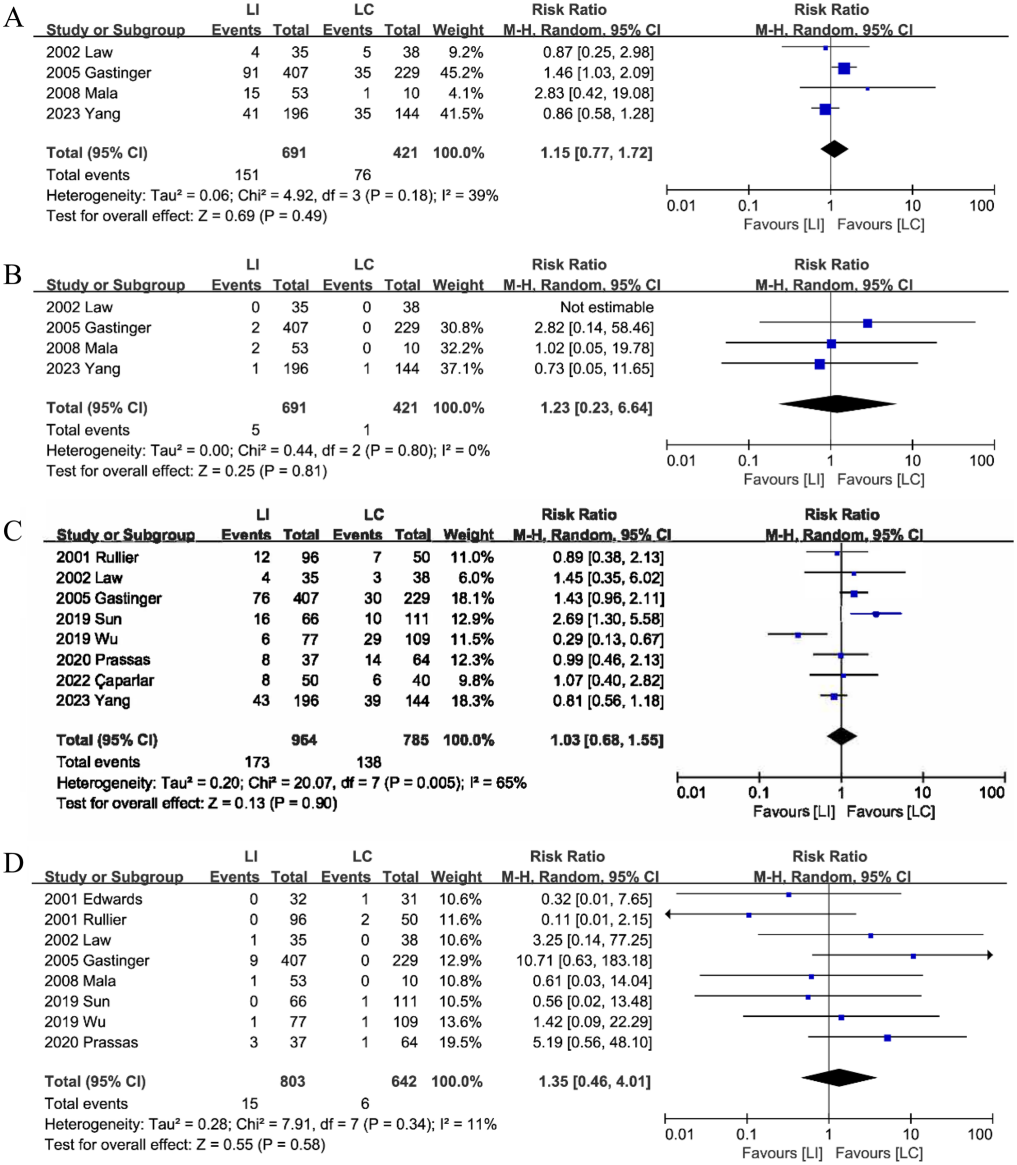
The morbidity (**A**), mortality (**B**),stoma closure related complication (**C**) and anastomotic fistula (**D**) after stoma closure between LI and LC groups

Between the two types of stomas, four studies [18, 20, 22, 24] compared morbidity following stoma reversal and found no statistically significant difference. (RR: 1.15; 95%CI: 0.77–1.72; P = 0.49; heterogeneity: P = 0.18, I^2^ = 39%) (Fig. 7A).

#### Mortality after stoma reversal

Four studies [18, 20, 22, 24] evaluated mortality rates between the LI and LC groups following stoma reversal. There was no discernible difference (RR: 1.23; 95%CI: 0.23–6.64; P = 0.81; heterogeneity: P = 0.80, I^2^ = 0%) (Fig. 7B) between the two group.

#### Stoma closure-related complication

Eight studies [15, 16, 18–22, 25] revealed specific postoperative problems. The findings indicated that there was no statistically significant difference between the two groups (RR: 1.03; 95%CI: 0.68–1.55; P = 0.90; heterogeneity: P < 0.01, I^2^ = 65%) (Fig. 7C).

#### Anastomotic fistula

Evidence from a combination of eight studies [15, 16, 19–24] suggests no significant differences regarding anastomotic fistula after stoma reversal between the LI and LC groups (RR: 1.35; 95%CI: 0.46–4.01; P = 0.58; heterogeneity: P = 0.34, I^2^ = 11%) (Fig. 7D).

#### Surgical site infections (SSI)

**Fig. 8:**
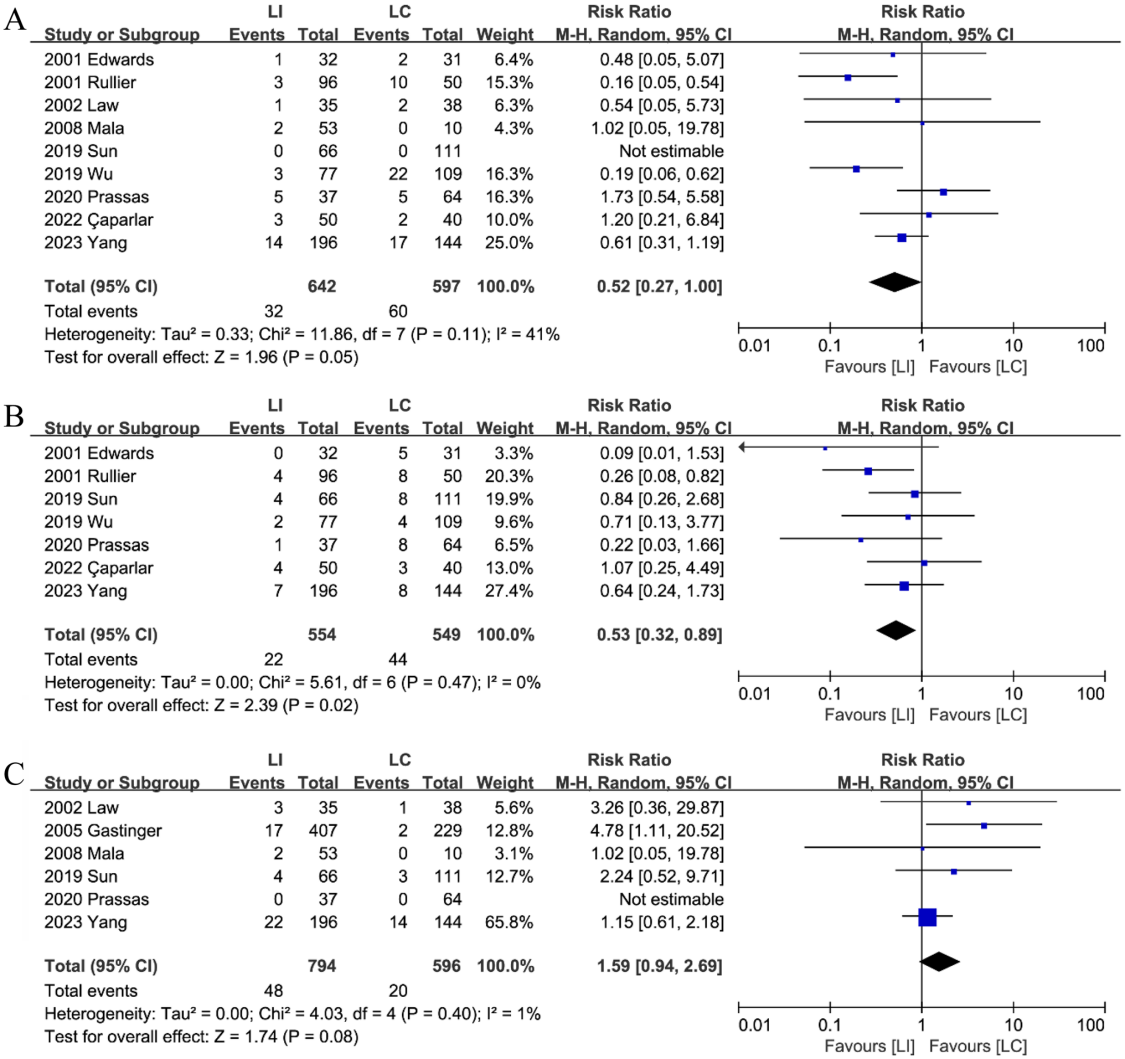
The Surgical site infections (**A**), Incisional hernia (**B**) and Ileus (**C**) after stoma closure between LI and LC groups

Nine studies [15, 16, 18–21, 23–25] published SSI data. Combining the data, the analysis revealed a statistically significant difference in the incidence of SSI between the LI group (5.0%, 32/642) and the LC group (10.1%, 60/597) following stoma reversal. This suggests that LI is less likely than LC to experience SSI (RR: 0.52; 95%CI: 0.27–1.00; P = 0.05; heterogeneity: P = 0.11, I^2^ = 41%) (Fig. 8A).

#### Incisional hernia

Seven studies [15, 16, 18, 19, 21, 23, 25] reported incisional hernias after stoma closure. Combined analysis showed that the patients from the LI group (4.0%, 22/554) had a lower incisional hernia when compared to patients from the LC group (8.0%, 44/549) (RR: 0.53; 95%CI: 0.32–0.89; P = 0.02; heterogeneity: P = 0.47, I^2^ = 0%) (Fig. 8B).

#### Ileus

Six studies [15, 16, 18, 20, 22, 24] reported data on ileus after stoma reversal. Combined analysis showed that a significantly higher incidence of ileus in the LI group (6.0%, 48/794) compared to the LC group (3.4%, 20/596) (RR: 1.59; 95%CI: 0.94–2.69; P = 0.08; heterogeneity: P = 0.40, I^2^ = 1%) (Fig. 8C).

#### Operation time

**Fig. 9:**
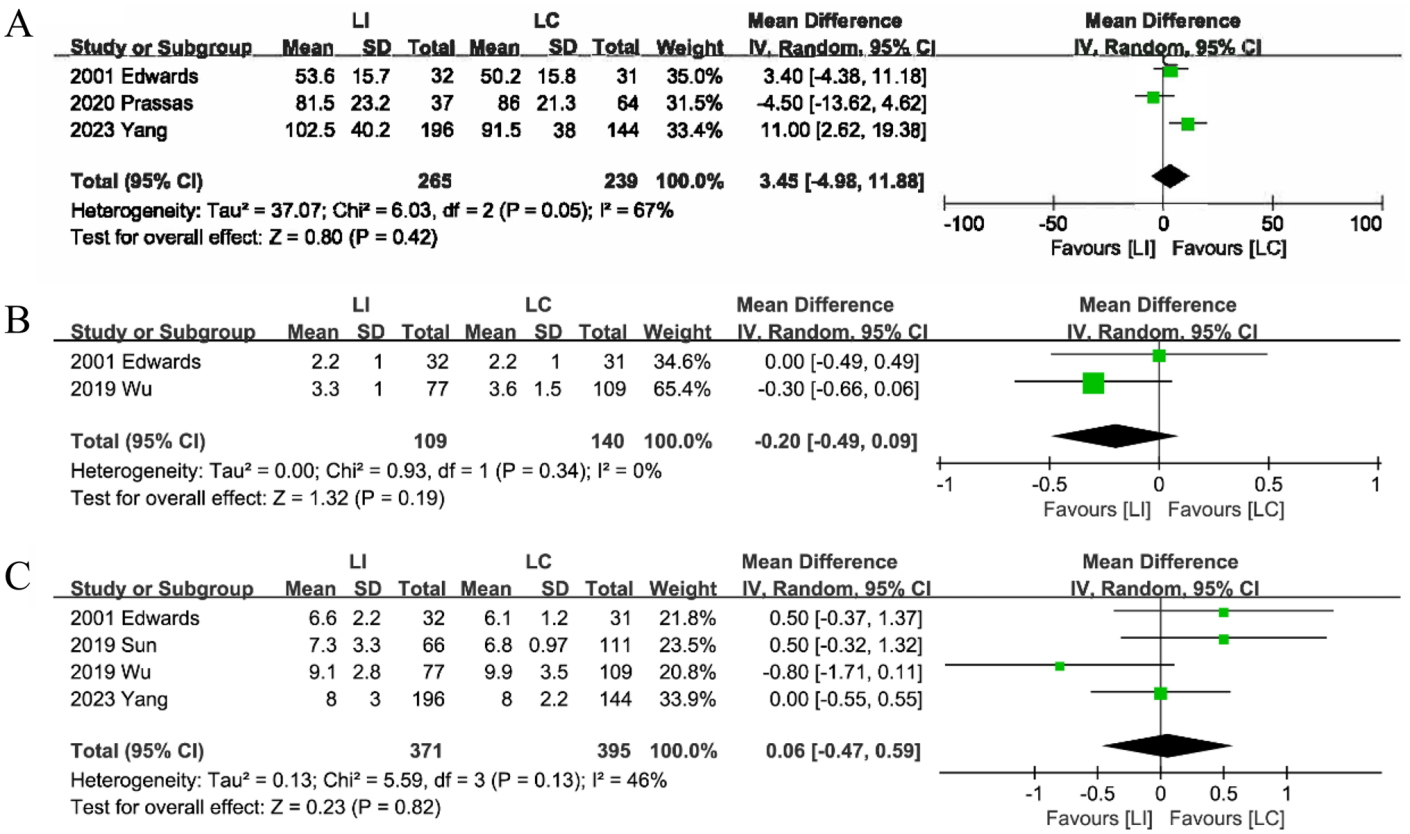
The Operation time (**A**), Time to first defecation (**B**) and Discharge (**C**) after stoma closure between LI and LC groups

Three studies [16, 18, 23] provided information on stoma closure operation time. The analysis showed no discernible difference between the LI and LC groups (MD: 3.45; 95%CI: -4.98–11.88; P = 0.42; heterogeneity: P = 0.05, I^2^ = 67%) (Fig. 9A).

#### Time to first defecation

The time to first defecation following stoma reversal was the subject of two studies [21, 23]. Thorough analysis revealed no significant differences between the LI and LC groups (MD: -0.20; 95%CI: -0.49–0.09; P = 0.19; heterogeneity: P = 0.34, I^2^ = 0%) (Fig. 9B).

#### Discharge

Combining data from four studies [15, 18, 21, 23] indicated no statistically significant difference between the LI and LC groups' hospital stays following stoma reversal (MD: 0.06; 95%CI: -0.47–0.59; P = 0.82; heterogeneity: P = 0.13, I^2^ = 46%) (Fig. 9C).

#### Sensitivity analysis

The trial by Yang et al. [18] (RR: 0.59; 95%CI: 0.17–2.08; P = 0.42; I^2^ = 0%) significantly affected the effect size of the stoma retraction**.** The study by Ruiller et al. [19] (RR: 1.88; 95%CI: 1.14–3.11; P = 0.01; I^2^ = 60%) and the study of Law et al. [20] (RR: 1.90; 95%CI: 1.11–3.27; P = 0.02; I^2^ = 62%) significantly affected the effect size of parastomal dermatitis after stoma formation. The study by Wu et al. [21] (RR: 2.43; 95%CI: 0.61–9.72; P = 0.21; I^2^ = 0%) significantly affected the effect size of the dehydration or electrolyte imbalance after stoma formation. The trial by Yang et al. [18] (RR: 2.82; 95%CI: 0.45–17.58; P = 0.27; I^2^ = 0%), Edwards et al. [23] (RR: 7.32; 95%CI: 0.90–59.22; P = 0.06; I^2^ = 30%) and Law et al. [20] (RR: 7.52; 95%CI: 0.96–59.22; P = 0.06; I^2^ = 28%) significantly affected the effect size of the high output**.** The study by Prassas et al. [16] (RR: 2.22; 95%CI: 0.85–5.80; P = 0.10; I^2^ = 0%) and Yang et al. [18] (RR: 2.20; 95%CI: 0.84–5.76; P = 0.11; I^2^ = 0%) significantly affected the effect size of the renal insufficiency after stoma formation. The study by Yang et al. [18] (RR: 1.44; 95%CI: 1.03–2.01; P = 0.03; I^2^ = 0%) significantly affected the effect size of the morbidity after stoma closure**.** Aditionally, the study by Yang et al. [18] (RR: 2.95; 95%CI: 1.21–7.20; P = 0.02; I^2^ = 0%) significantly affected the effect size of the ileus after stoma closure. The study by Rullier et al. [19] (RR: 0.64; 95%CI: 0.36–1.14; P = 0.13; I^2^ = 0%) affected the effect size of incisional hernia after stoma closure. Due to the small number of patients included, the subgroup analysis was limited.

#### Publication Bias

**Fig. 10:**
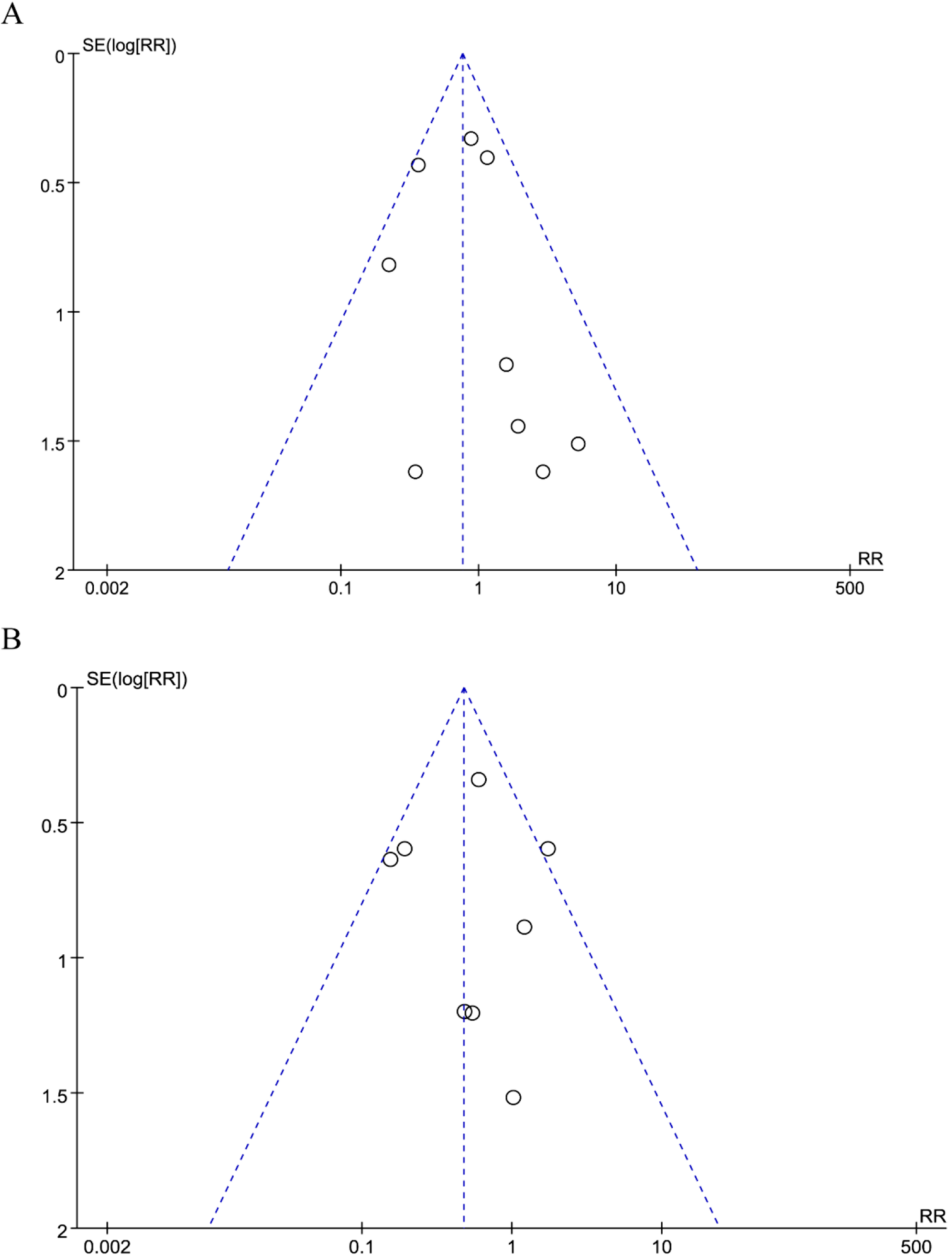
The funnel plot of **A** parastomal hernia. **B** Surgical site infections rate

No significant publication bias regarding parastomal hernia after stoma formation or surgical site infections after stoma closure was observed in the funnel plots (Fig. 10).

## Discussion

The preventive defunctioning stoma was performed to reduce reoperation morbidity and mortality in high-risk anastomose [26, 27]. Nevertheless, the optional type of protective ostomy remains a subject of debate. The choice of LI and LC for the stoma is somewhat arbitrary for surgeons. This meta-analysis was conducted to evaluate perioperative complications and complications of stoma closure with that of LI or LC formation during the early stages of anterior resection for rectal cancer.

First, the current study showed no discernible variations in morbidity and mortality among the early stages of anterior resection for rectal cancer, stoma creation, and stoma reversal. Both choices of ostomy are safe. Results of the present meta-analysis show that patients in the LI group had a lower incidence of stoma prolapse and retraction than those in the LC group. Prior investigations have also documented lower rates of stoma retraction rates in ileostomy compared to colostomy [28, 29]. Consistent with our findings, earlier meta-analyses have shown a 2% lower incidence of stoma prolapse in the LI group compared to an 11% prolapse rate in the LC group [30]. This discrepancy could be attributed to the higher number of fascial defects resulting from transverse colostomy and the heavier content in the colon than in the ileum. Stoma retraction was frequently observed in female patients who underwent a protective colostomy because of the appearance of a skin fold at the waist over the upper abdomen due to a loose and floppy abdomen. Furthermore, our research revealed that the LI group experienced higher rates of renal insufficiency and high-output problems associated with their stoma than the LC group. One hypothesis is that the contents of ileum are more concentrated and diluted than those of the colon. According to reports. Up to 31% of small bowel stomas involve ileostomy, which frequently results in excessive output [31–33]. Complications such as dehydration, involving salt and water depletion, and renal impairment, can arise if the ileostomy output remains excessive [34–36]. Acute kidney injury (AKI) has been reported in 25% of patients receiving chemotherapy for ileostomy. AKI negatively affects adjuvant therapy, disease-free survival (DFS), and OS. Therefore, close attention should be paid to fluid balance and electrolyte management in patients with some degree of impaired renal function. The choice of ileostomy requires close monitoring and postoperative stoma care. A higher prevalence of peristomal skin irritation dermatitis is caused by the alkaline effluent produced by ileostomies, which is rich in proteolytic enzymes and irritates the exposed peristomal skin. Moreover, certain observational studies have shown that patients who underwent ileostomy are more prone to diarrhea, electrolyte abnormalities, and irritating dermatitis than those who underwent colostomy [37, 38]. However, our study found no significant difference in the incidence parastomotic dermatitis between the LI and LC groups. One possible explanation is the variation in the definitions of skin irritation used in different studies.

In contrast to LC, LI has a higher rate of ileus but a lower incidence of SSI and incisional hernia, according to our research. Incisional hernia is more common in the colostomy than in ileostomy [39, 40]. The significant fascial defect caused by the transverse colostomy may have caused of the higher incidence of incisional hernia in the LC group following stoma closure. Compared to the LI group, the LC group had a significantly higher incidence of SSI. The cleaner intestinal environment of the ileum compared to the colon could be the reason for this difference. Ileostomy has been shown to reduce the incidence of systemic infections, such as sepsis, in addition to local wound infections [41]. During surgery, incision protection devices can successfully shield the incision and prevent wound infection [42].In the present study, the definition of ileus is different. Two studies [15, 20] reported intestinal obstruction and ileus [18], while other studies [16, 22] reported ileus. Therefore, the result regarding ileus require further consideration.

Several factors have been associated with AL, such as advanced age, BMI, male sex, ASA, and tumor size [43, 44]. In terms of BMI, sex, and ASA [28], the present review did not reveal any statistical differences between LI and LC. Considering that a significant percentage of patients are overweight or obese and that the elderly population is more susceptible to rectal cancer [1, 45], they often have a high risk of AL, making their choice of stoma prudent. However, older adults have relatively weak abdominal walls, which can increase the risk of stoma prolapse, colostomy development, and incisional hernias following colostomy reversal. Furthermore, individuals who are obese typically have shorter mesentery lengths and thicker subcutaneous layers, making colostomy more challenging. Therefore, Rosen et al. [46] showed that the usage of an ileostomy is recommended for patients who are obese, in whom adequate mobilization of the transverse colon is not possible. Furthermore, because the colostomy was located in the upper abdomen and somewhat farther from the radiation area, it would be a logical choice to perform the procedure if the patients needed a colostomy to receive postoperative radiation.

In addition to the abovementioned elements, it is important to highlight that the distal intestine lacks fecal stream stimulation for several months following stoma creation, which might affect physiology, particularly in the case of ileostomy. This stimulation involves mechanical forces, microorganisms, and microbial metabolites [47]. However, further studies are required to explore the relevant pathophysiological changes. When selecting a stoma type, patient's lifestyle choices and quality of life should also be taken into account. For instance, the patient's belt setting may clash with the location of the distal ileostomy in the lower abdomen, and heavy body hair in some patients may affect the effectiveness of the sticker chassis. Additionally, the odor of colon stoma secretions can be bothersome for some individuals. Therefore, it is crucial to consider each patient’s unique needs, including their quality of life, sex, age, physiological state, body mass index (BMI), presence of obesity, and the timing of their treatment, when deciding whether to opt for protective colostomy or ileostomy. By taking these factors into account, a more informed and tailored decision regarding the type of ostomy procedure that is most suitable choice for each patient can be made.

Our analyses have some limitations. First, the results should be interpreted cautiously due to the inclusion of only two RCTs and eight cohort studies, with insufficient patients across these investigations. Consequently, the meta-analysis was weak, as expected event, such as parastomal dermatitis and renal insufficiency were low. Additionally, certain parameters, such as the operation time for stoma creation, and the size of the incision during stoma closure, should be compared between the two groups, whereas the included trials did not make the comparison. Moreover, the choice of surgical technique for stoma placement is a significant factor that could affect the duration of stoma formation and the likelihood of associated complications. In contrast to conventional fixation to the peritoneum and anterior rectal sheath, some surgeons advise the one-stitch method for creating protective loop ileostomies, which has the advantage of saving operating time [48]. Fewer studies have reported new methods to create loop colostomies. Finally, the high heterogeneity observed in this study for stoma-related complications, stoma stricture, parastomal dermatitis, stoma closure-related complications, and stoma closure operation time may be related to differences in the included study designs (including RCTS and retrospective studies), variations in the definitions of associated complications, and discrepancies in the length of the stoma reduction interval.

## Conclusion

Our study suggests that compared to LC, LI is associated with a higher incidence of dehydration or electrolyte disturbance, high-output, and renal insufficiency, while demonstrating a reduced incidence of stoma prolapse and retraction, SSI, and incisional hernia. These findings suggest the use of a prophylactic diverting loop ileostomy during anterior rectal resection for rectal cancer. Naturally, patients with renal failure require additional monitoring. In addition, other individual patient characteristics such as obesity, physiological status, requirement for radiotherapy, and patient quality of life should be considered. Finally, further excellent prospective multicenter studies with large randomized controlled sample sizes are needed to provide additional confirmation of these findings.

## Data Availability

No datasets were generated or analysed during the current study.
